# Interest in neurology during medical clerkship in three Nigerian medical schools

**DOI:** 10.1186/1472-6920-10-36

**Published:** 2010-05-20

**Authors:** Emmanuel O Sanya, Olugbenga E Ayodele, Timothy O Olanrewaju

**Affiliations:** 1 Neurology unit, Department of Medicine, University of Ilorin Teaching Hospital, PMB 1459, Ilorin Kwara State, Nigeria; 2 Department of Medicine, Ladoke Akintola University of Technology, Osogbo, Nigeria; 3 Renal Unit, Department of Medicine, University of Ilorin Teaching Hospital PMB 1459 Ilorin, Kwara State, Nigeria

## Abstract

**Background:**

This study sought to ascertain perception of Nigerian medical students of neurology in comparison with 7 other major medical specialties. To also determine whether neurology was the specialty students consider most difficult and the reasons for this and to appraise their opinion on how neurosciences and neurology were taught in their different universities.

**Methods:**

Self-administered questionnaires were used to obtain information from randomly selected clinical students from 3 medical colleges in Nigeria (University of Ibadan, Ibadan; University of Ilorin, Ilorin; Ladoke Akintola University of Technology, Osogbo).

**Results:**

Of 320 questionnaires sent out, 302 were returned given 94% response rate. Students felt they knew neurology least of all the 8 medical specialties, and were not confident of making neurological diagnoses. About 82% of the students indicated they learnt neurology best from bedside teaching, followed by use of medical textbooks. Close to 15% found online resources very useful for learning neurology and 6% indicated that group discussion was quite useful in the acquisition of knowledge on neurology. Histology and biochemistry were the preclinical subjects participants opined were least useful in learning neurology. The most frequent reasons students felt neurology was difficult were problems with understanding neuroanatomy (49%), insufficient exposure to neurological cases (41%), too many complex diagnoses (32%) and inadequate neurology teachers (32%).

**Conclusions:**

Nigerian medical students perceived neurology as the most difficult medical specialty and are not interested in specializing in it. Neurology education could be improved upon by provision of more bedside tutorials and increased availability of online resources to enhance learning. There is need to emphasize increased frequency of small group discussions amongst students so that they will be used to teamwork after graduation.

## Background

The term "neurophobia" was originally coined by Jozefowicz to describe the fearful perception of neurology and neurological science by medical students [1]. However, the phenomenon has been a long-standing problem. Reported signs of neurophobia vary from confusion to display of intimidation, boredom and impatient desire for the class to end. Students with neurophobia during clinical posting are eager for the posting to come to an end [1]. Fear of neurology and avoidance of neurologic examination is very common amongst general practitioners (GP) [2,3]. In the survey by Thapar *et al*, GP rated themselves low in confidence and in caring for neurological disorders [2]. Patients have also corroborated this view that non-specialist doctors' show lack of confidence and are unwilling to manage neurological diseases [4].

Recently, there has been a change in the epidemiological pattern of diseases in most countries and neurological disorders are at the centre of this transition [5-7]. The number of acute and chronic neurological diseases seen in most out-and in-patient services are on the increase [8-10]. For example more cases of strokes, dementias and neurodegenerative disease have been reported [6,8] which may be a reflection of lifestyle changes in most communities toward western culture. The increased contribution of neurological diseases to the global burden of diseases has made the World Health Organization (WHO) to declare disorders of the neurological systems as a major public health problem [9]. In view of the fact that majority of these neurological disorders are often first seen by the GPs before referral to the specialist [10,11], it is pertinent that GPs, especially those practicing in developing countries with limited facilities brace themselves for the challenges of managing patients with neurological disorders before referral.

Reasons that have been adduced as to why students and doctors alike do not show interest in neurology include short duration of neurologic education, unfocused education and training, and the separation of basic neurosciences and clinical studies at medical schools [1,4,12]. This study therefore, sets out to ascertain the level of interest of medical students in neurology in comparison with seven other major medical specialties in three Nigerian universities. The study also sought to answer whether perception of neurology as a difficult subject poses a serious challenge to students when they try to learn and practice the subject and whether it influences their choice of specialization.

## Methods

### Study design and Study sample

This is a cross sectional study involving medical students from three generation of Nigerian universities. A medical school was chosen from the first generation (University of Ibadan), second generation (University of Ilorin) and third generation (Ladoke Akintola University of Technology) universities in Nigeria. Study participants were clinical students in 4^th^, 5^th ^and 6^th ^year of medical training. Ethical approval for study was obtained from ethics committee of our institution. Questionnaires were distributed to the students immediately after a lecture and the students were asked to fill the questionnaires without indicating their names. Verbal consent was obtained from each student after the aim and purpose of the study had been explained to the students.

### Instruments

A self-administered questionnaire was designed based on previous similar studies [4,13]. The questionnaire contains 10 questions which were divided into two sections. The first section was designed to determine the depth of students' knowledge of neurology. The second section addressed why students felt neurology was difficult and possible solution on ways of improving the teaching of neurology and neurosciences. The first section consisted of four questions which addressed students' perception of their knowledge of neurology in comparison with seven other medical specialties; their level of confidence in making neurological diagnoses; their level of confidence in managing neurological conditions; and lastly the setting students felt they learnt neurology most in school. In the second section, students were asked to rate teachings of neurology and neurosciences in the preclinical and clinical years. Furthermore, they were asked how relevant they felt the preclinical subjects were when applied to clinical clerkship in neurology. Students were asked to rate their level of interest in four neuroscience disciplines taught in our medical schools (neurology, neurosurgery, psychology and psychiatry). There were two open questions in this section. The first question asked participants to give reasons why neurology was perceived difficult and second question asked the students to proffer probable solutions. Students were finally asked about the likelihood of specializing in neurology after their medical education.

Responses were graded on a maximum scale of 3 or 5 depending on the structure of the question (1 was the lowest possible score and 3 or 5 the highest). For example, responses to questions on the usefulness of preclinical subjects to clinical clerkship and why neurology was considered to be difficult had a maximum score of 3 (not a contributor-1, a minor reason-2, major reason-3). The responses to questions on how knowledgeable participants were of various medical specialties had maximum rating of 5 (very difficult-1; moderately difficult-2; mildly difficult-3; easy-4; very easy-5).

### Data analysis

Data were analyzed with the Statistical Package for the Social Sciences version 11 (SPSS Inc). Frequency tables were generated for the variables. Means and standard deviations were determined. Mean scores of responses were calculated and student t-test was used to analyze the differences in mean values of responses. P value less than 0.05 was considered statistically significant.

## Results

Out of 320 questionnaires sent out, 302 were returned giving a response rate of 94%. One hundred and eighty-one participants (60.0%) were males and 121 (40.0%) were females. Distribution of participants who returned the questionnaire showed that 28% were in the 4^th ^year, 15% in the 5^th ^year, and 51% in the 6^th ^year.

### Section 1

**Figure 1:**
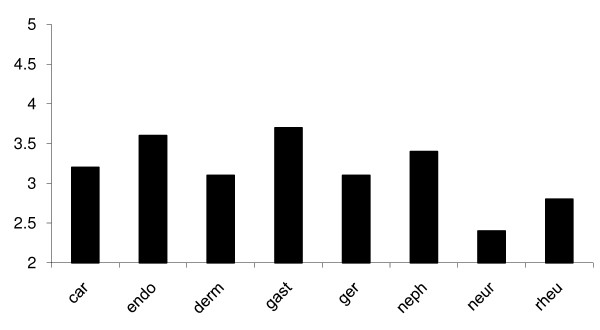
**Self-rated knowledge of neurology in comparison to other subjects**. **Keys**: car-cardiology; endo-endocrinology; derm-dermatology; ger-gerontology; gastgastroenterology; neph-nephrology; neur- neurology; rheu- rheumatology

**Figure 2:**
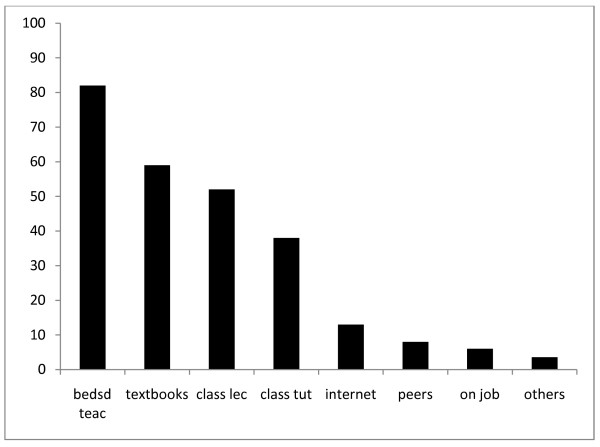
**Where students learn neurology most**. **Keys**: bedsd teach- bedside teachings; class lec- classroom lectures

The students were asked to rate their level of knowledge of 7 medical specialties in comparison with neurology. Participants rated neurology least, followed by rheumatology and geriatrics while gastroenterology and endocrinology were rated highest (Figure 1). The difference was statistically significant (p < 0.05). A total of 296 students responded to the question on how easy it was to make neurological diagnoses. Students were not very confident to make diagnosis of neurological disorders. Close to 46% of them believed making neurologic diagnosis is moderate to very difficult, while 8% opined it was relatively ease (Table 1). With regards to their opinion on management of neurologic disorders, 16% of participants felt it was moderate to very difficult, while 30% gave no response. Students were also asked the setting they learnt neurology most in medical college. Close to 82% indicated they learnt the most from bedside teaching and 59% from use of medical textbooks. Other common ways by which the students felt they learnt neurology were classroom lectures (38%) and from online resources (13%). Only 6% indicated they learnt neurology from peers during group discussion (Figure 2).

**Table 1:** Responses to questions on diagnosis and management of neurologic disorder

Questions	Frequency of responses, n = 302 (%)
**Diagnosis of neurological disorder is**	
	
Very difficult	39(13%)
Moderately difficult	100(33%)
Mildly difficult	130(43%)
Easy	24(8%)
Very easy	3(1%)
No response	6(2%)
	
**Management of neurological disorders is**	
	
Very difficult	12(4%)
Moderately difficult	36(12%)
Mildly difficult	130(43%)
Easy	36(12%)
Very easy	3(1%)
No response	91(30%)

### Section 2

**Figure 3:**
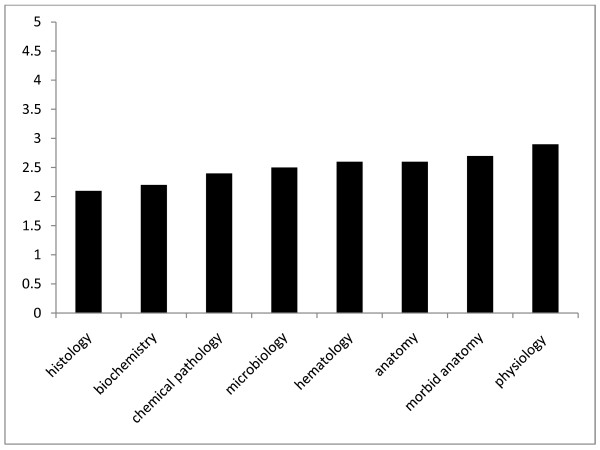
How helpful are basic neuroscience courses to clinical clerkship

Of the basic medical science subjects, this group of students found histology and biochemistry least useful in the acquisition of knowledge in neurology. They found physiology and morbid anatomy most helpful in learning neurology, with mean score (SD) of 2.9 ± 0.5 and 2.7 ± 0.4, respectively (Figure 3). The most frequent reasons why medical students felt neurology was difficult were: difficulty with neuroanatomy teachings (49%), occurrence of complex diagnoses (35%), insufficient teaching (32%) and inadequate neurology teachers (32%) [Table 2]. About 25% of students had difficulty with neurologic examination. Responses to question on quality of teaching of neuroscience courses received in preclinical and clinical year had a maximum score of 5. There was a significant difference in the way students rated the teaching of basic neuroscience subjects of preclinical classes and clinical subjects. Lectures received in the clinical classes were rated higher than the preclinical ones with mean score (SD) 3.33 ± 0.6 vs. 2.49 ± 0.8, p < 0.05. The responses of the students on the level of interest in the four-neuroscience disciplines showed that a higher proportion of students were interested in psychiatry (60%) and psychology (51%) than neurosurgery (46%) and neurology 49% (p < 0.05). Only 4% of the students indicated they would like to become a neurologist upon completion of their undergraduate study. There were 329 suggestions on ways to improve neurology education and these were summarized under 8 main themes (Table 1). The frequent suggestion was the need for more neurology teachings with emphasis on clinical and bedside teachings, followed by provision of more teaching aids and models. The third most common suggestion was the demand for increase in the number of neurology lecturers in the faculty.

**Table 2:** Responses (multiple) to open questions

Questions	Responses
Why is neurology difficult?	
	
Trouble with understanding neuroanatomy	148(49%)
Inadequate model/aids	137(45%)
Limited exposure to cases	124(41%)
Complex diagnoses	107(35%)
Insufficient teaching	97 (32%)
Inadequate teachers	97 (32%)
Trouble with neurosciences	83(28%)
Difficult neurology examination	76(25%)
Others	18(6%)

How can neurology lecture be improved upon?	
Provide more teaching	152(52%)
More aids/models	98(32%)
More teachers	59(20%)
More clinical rotation	45(15%)
More tutorial	14(5%)
Reduce student population	9(3%)
Others	18 (6%)

## Discussion

The findings of this study showed that Nigerian medical students perceived neurology to be the most difficult of all medical specialties. This is evident by respondents' rating of their knowledge of neurology least of all the eight medical disciplines. The subjects students felt they were most knowledgeable about were gastroenterology and nephrology. It was surprising that participants rated their knowledge of neurology below geriatrics and rheumatology, two specialties that are just being developed in most medical schools in Nigeria. It is presently difficult to give a reason for such a response. Participants felt they learnt neurology most during ward rounds. This observation underscores the effectiveness of traditional clerkship and bedside teachings whereby signs are demonstrated to clinical students. Other ways by which the participants learnt neurology most were through seminars, bedside teachings during tutorial sections and use of medical textbooks. Our result compares with those of Schon *et al *from UK [3] and that of Flagan *et al *in Ireland [13]. In both studies, students cited gastroenterology as the subject they were most knowledgeable in and considered neurology to be the most difficult of all medical specialties. The results of these studies and ours were on students' opinion and may not truly reflect the depth of their knowledge of these disciplines. Nevertheless, the finding is noteworthy and may require further study to relate their actual test scores with their opinions.

Of all the preclinical courses, histology and biochemistry were indicated to be least helpful for learning neurology. These 2 courses are usually taught early in medical schools in Nigeria. It may be that these subjects were taught in an unrelated manner so that it gave an impression they were unimportant in the latter part of their training [3]. Part of the reasons why the students found neurology particularly difficult is that they had trouble in understanding neuroanatomy, a situation made more complex by availability of limited number of teaching aids. They also had problem with too many complex diagnoses made by neurologists. Difficulty with understanding neuroanatomy is particularly a recurring theme from several similar studies [13]. The main reason that had been adduced for this is the abstract manner in which neuroanatomy had been taught, and there are opinions that this manner of teaching needs to be changed. This abstract method requires visuo-spatial activity, a function of the right cortex which is best suited for Faculty of Arts students rather than medical student. Even though the structure and functions of the entire central nervous system may be complex than most body organs, neurology is not so complicated if taught from basics and in a simplified manner in relation to common diseases [3].

Another interesting finding of this study is that only 6% of the participants indicated they learned neurology from their colleagues. It could be inferred that only few medical undergraduates in this study practice small group discussions with their peers. This observation is pertinent because doctors often do not practice in isolation, but rather as a team with colleagues and other health care workers. Thus, these students might find it difficult to work with colleagues and other members of health team after graduation. A study that had looked at ways to improve medical education had suggested introduction of small group discussion. This is likely to encourage team work, increase students' comprehension and make them lifelong learner [14].

Over 15% of our participants found online resources very useful for learning neurology. This proportion is more than 1% of student reported from Ireland [13] but less than the proportion of US students that reported that online resources are veritable tool for neurology education [15]. A recent study on alternative ways to facilitate learning of basic and clinical neurology in USA found use of e-textbook as a good alternative [16]. The conclusion of the study was that after 6 years of introduction of e-textbook and online resources, neurology education was made easier with marked increase in student's satisfaction with the subject [16].

Unfocused teaching in neurology is another reason that has been found to be responsible for why doctors and medical students are neurophobic. It is, therefore, important that the teaching in basic neuroscience and clinical neurology must be more effectively integrated. Neurologists in the past do pride themselves during ward rounds about making complex diagnoses of rare disorders and unusual syndromes more than any other medical specialties [12]. This view was corroborated by responses of more than a third of our students' responses that they had problems with the complex diagnoses made in this specialty. To overcome this view, teaching of clinical neurology should focus more on common disorders and this should be done using simplified terminologies. Over 52% of the students proposed provision of more teachings in neurology and increase in number of neurology teachers as ways of improving neurology education. We believe this suggestion is crucial to improvement of neurology education in Nigeria. The work of Ridsdale *et al *from UK supported this view [12]. In that report, after neurology had been taught for 13 weeks and mostly in consultant-led teachings, students' understanding and rating of neurology was comparable to other medical disciplines, while their skill of neurology examination was greatly improved upon [12]. The use of small group bedside teachings that the students found most beneficial will also facilitate better understanding of the subject. Therefore, provision of more focused teaching along with increased duration of neurology course will help in improving the interest of students in neurology. Furthermore, there is a critical need to encourage specialization in this discipline based on the recent disturbing report that the number of neurologist in most African countries is very few with estimate of 0.03 neurologists per 100,000 populations [17]. Nigeria with current estimated population size of 140 million has only 50 registered neurologists [18]. More neurologists are needed in developing countries as practitioners to improve neurological practices and also as educators and health policy advisers and advocates. Unfortunately, only 4% of the study participants were interested in specializing in neurology. Thus, the teaching of clinical neurology will still have to depend on non- neurology specialists in most medical schools.

## Conclusions

In conclusion, results of this study confirmed that medical students perceived neurology as a difficult subject than other medical subjects. This is likely to be the view of majority of medical undergraduates in the country because the study participants were drawn from three medical schools representing three generations of Nigerian universities. Reasons why the students found neurology difficult and uninteresting include difficulty in understanding neuroanatomy, lack of teaching aids/models and poor teaching of neurosciences subjects. This lack of interest in neurology could be a hindrance to the choice of neurology as a specialty. In order to stem the tide of the burden of neurological diseases in Nigeria, there is a need for capacity building in neurology and this can be achieved by improving on the teaching of neurosciences and neurology in order to stimulate the interest of students in specializing in this field. In addition, introduction of e-textbooks and online resources may likely facilitate better learning of neurology.

## Competing interests

The authors declare that they have no competing interests.

## Authors' contributions

EOS and OEA conceived the study. EOS coordinated the study, analyzed the data and drafted the initial manuscript. The collection of data and review of initial manuscript were carried out by EOS, OEA and TOO. All authors read and approved the final manuscript.

## Pre-publication history

The pre-publication history for this paper can be accessed here:

http://www.biomedcentral.com/1472-6920/10/36/prepub
